# Towards a Rational Screening Strategy for Albuminuria: Results from the Unreferred Renal Insufficiency Trial

**DOI:** 10.1371/journal.pone.0013328

**Published:** 2010-10-13

**Authors:** Arjan van der Tol, Wim Van Biesen, Francis Verbeke, Guy De Groote, Frans Vermeiren, Kathleen Eeckhaut, Raymond Vanholder

**Affiliations:** 1 Renal Division, Department of Internal Medicine, University Hospital Gent, Gent, Belgium; 2 CRI Laboratory, Gent, Belgium; 3 Occupational Health Care Adhesia, Gent, Belgium; Yale University School of Medicine, United States of America

## Abstract

**Background:**

There remains debate about the screening strategies for albuminuria. This study evaluated whether a screening strategy in an apparently healthy population based on basic clinical and biochemical parameters could be more effective than a strategy where screening for albuminuria is performed unselectively.

**Methodology/Principal Findings:**

The Unreferred Renal Insufficiency (URI) Study is a cross-sectional study on the prevalence of metabolic risk factors in Belgian workers, volunteering to be screened during a routine yearly occupational check-up. Subjects (n = 295) with treated hypertension, known diabetes, treated dyslipidaemia, cardiovascular and renal disease were excluded. Among 1,191 apparently healthy subjects, 23% had unknown hypertension, 13% had impaired glucose tolerance, 15.4% had normoalbuminuria, 4.2% had microalbuminuria and 0.4% had macroalbuminuria. Subjects with resting heart rate ≥85 bpm, plasma glucose ≥5.6 mmol/L and blood pressure ≥140/90 mmHg were associated with albuminuria of any degree. A strategy where only subjects with at least one of these risk factors (n = 431) were screened for albuminuria, would identify all subjects with macroalbuminuria (5/5), 64% of those with microalbuminuria (32/50), and less than half of those with normoalbuminuria (81/183). An alternative strategy whereby subjects were first screened for presence of albuminuria, and additional cardiovascular risk factors were only measured in subjects positive for albuminuria (n = 238), would identify only 27% (118/431) of the subjects with additional and potentially modifiable cardiovascular risk factors. On the other hand, half of the subjects in this study with albuminuria (120/238, of which 102 had normoalbuminuria), had no additional cardiovascular risk factor at all.

**Conclusions:**

Screening an apparently healthy population directly for albuminuria will result in a high percentage of false positives, mostly measured in the normal range. Screening for microalbuminuria and macroalbuminuria based on presence of additional, potentially modifiable risk factors appears to be more beneficial. **Trial registration** 2006 NCT00365911

## Introduction

The number of patients with end stage renal disease (ESRD) in need of renal replacement therapy has dramatically increased over the last decades [1]. A substantial part of this increase is attributable to the rising prevalence of diabetes and/or hypertension [2], [3], [4]. Microalbuminuria seems to be an important predictor of progressive renal disease and end stage renal disease in diabetic and hypertensive subjects [5], [6]. It has been demonstrated that in these populations, preventive measures can delay the evolution to macroalbuminuria and potentially also the progression to renal failure [7], [8]. Consequently, it is advised to screen these high risk subjects with diabetes and hypertension, in order to identify and eventually treat those at risk for progressive renal disease. Extending this line of reasoning, some authors advocate to screen also low risk groups, or even the general population, arguing that most persons with albuminuria and/or reduced eGFR (<60 ml/min/1.73 m^2^) are asymptomatic [9]. Van der Velde et al e.g. found that 45% of subjects with microalbuminuria were younger than 55 years and had no hypertension or diabetes [9]. It is debated whether such a large scale screening project should be advocated [10], [11]. Concerns are not only the cost of screening itself, but more importantly, the risk and the cost of treating false positive subjects [12]. In the study by van der Velde et al, out of 40,854 subjects screened, 7.8% (n = 3,200) had at least microalbuminuria, but only 45 of those developed ESRD over a 9 year follow up period [9]. In addition, effective screening presumes that an intervention to alter the course of the disease is available [13], which is not the case if the subject only has albuminuria and no other modifiable risk factor. It would therefore be very useful to develop a screening strategy based on additional, modifiable, risk factors to enhance the yield of screening without having too much false positives, and to make interventions possible.

In this study, we used data collected in an apparently healthy working population, aged 17 to 65 years, to evaluate which easily obtainable parameters or combinations of them are associated with albuminuria, and whether using these risk factors can be of help to increase the effectiveness of a screening program.

## Methods

### Objectives

The primary aim was to determine the prevalence of different levels of albuminuria, some (cardiovascular) risk factors and their associations, to develop a rational screening strategy for albuminuria in the healthy population.

### Participants

The Unreferred Renal Insufficiency (URI) is a cross sectional study that included only Caucasian workers (n = 1,486) who presented at a routine yearly occupational check-up between January 2007 and December 2009, in Belgium. The presence of more than 100 leukocytes/µl (2.2%), and/or more than 50 erythrocytes/µl (2.4%) in the urinary sediment were considered as confounders for reliable measurement of urinary albumin; these subjects and subjects with incomplete data (1.8%) were consequently excluded from further analysis. Subjects with known comorbid conditions or risk factors of which it is well established that they could affect albuminuria, such as diabetes (1.2%), cardiovascular disease (0.3%), renal disease (0.4%), subjects on antihypertensive drugs (8.3%) and on lipid lowering drugs (3.3%) were also excluded, leaving a cohort of 1,191 apparently healthy subjects for analysis.

### Description of procedures

All subjects were investigated during their yearly check up by their occupational physician. Body weight was recorded to the nearest 0.1 kg and height was measured to the nearest centimeter. Waist circumference was measured by trained nurses following recommendations by WHO [14]. Body mass index (BMI) was calculated as body weight in kg divided by height^2^ (kg/m^2^). Blood pressure and resting heart rate were measured in sitting position by a calibrated electronic device (OMRON®). A questionnaire about current cigarette smoking, physical activity and prescribed medication was taken by an occupational physician in each participant. A random blood and urine spot specimen was collected and analyzed on the same day in one central laboratory (no frozen samples). Urinary albumin was measured by an immune turbidimetric method with an inter-assay coefficient of variation of 11.2% at a mean level of 82 g/L and an inter-assay coefficient of variation of 4.9% at a mean level of 580 g/l. Serum creatinine was analyzed by a colorimetric assay (compensated Jaffe reaction), calibrated by Isotope Dilution Mass Spectrometry (IDMS), with an inter-assay coefficient of variation of 1.75% at a mean level of 104 µmol/l mg/dl (Roche). Serum CRP was measured with an immune turbidimetric method with an inter-assay coefficient of variation of 4.6% at a mean level of 3.2 mg/l and inter-assay coefficient of variation of 2.5% at a mean level of 5.5 mg/l.

### Definitions

Gender specific urinary albumin creatinine ratio (uACR) cutoff values as proposed by Warram et al. were used because men have a higher urinary excretion of creatinine than women due to higher muscle mass. Normoalbuminuria was defined as uACR: 0.6–1.8 mg/mmol (5–16 mg/g) in men and uACR 0.8–2.7 mg/mmol (7–24 mg/g) in women, microalbuminuria was defined as uACR 1.9–27 mg/mmol (17–249 mg/g) in men and uACR 2.8–39 mg/mmol (25–354 mg/g) in women, macroalbuminuria was defined as uACR ≥28 mg/mmol (250 mg/g) in men and uACR ≥40 mg/mmol (355 mg/g) in women [15]. The Modification of Diet in Renal disease (MDRD) equation was used to assess the estimated GFR: eGFR = 30849 × standardized S_cr_^−1.154^ × age^−0.203^ × 1.212 [if black] × 0.742 [if female][16]. Impaired glucose tolerance (IGT) was defined as a plasma glucose level ≥5.6 mmol/L [17]. Hypertension was defined as diastolic blood pressure ≥90 mmHg and/or systolic blood pressure ≥140 mmHg. Obesity was defined as BMI >30 kg/m^2^. Abdominal adiposity was defined according the National Cholesterol Education Program (NCEP) criteria: waist circumference >102 in men and >88 cm in women. Hypercholesterolemia was defined as serum total cholesterol >6.5 mmol/L.

### Ethics

A written informed consent was obtained from all participants. The Ethics Committee of University Hospital Ghent approved the study (2006-038).

### Statistical methods

SPSS 15.0 was used for all calculations. Results are presented as percentages or as mean ± standard deviation. The baseline characteristics of groups were compared by use of ANOVA: post-hoc Scheffé test (continuous variables), Kruskal-Wallis (continuous variable with a skewed distribution) and a Chi-square test (categorical variables). Ordinal regression analysis was performed to select the associated risk factors with different categories of albuminuria, in a random selection of 50% of the subjects. The significant continuous variables were dichotomized. These risk factors were validated in the other 50% of the sample. The prevalence and test characteristics, of normo-, micro- and macroalbuminuria were calculated, if at least one of these risk factors was present. The sensitivity was defined as the number of subjects with true-positive test results divided by the total number of subjects with albuminuria. The specificity was defined as the number of true-negative test results divided by the total of number of subjects without albuminuria. The positive predictive value was defined as the number of true-positive test results divided by the total number of positive test results. The negative predictive value was defined as the number of true negative test results divided by the total number of negative test results. The positive likelihood ratio was defined as sensitivity divided by 1-specificity; the negative likelihood ratio was defined as 1-sensitivity divided by specificity.

## Results

Our cohort (n = 1,191) of apparently healthy subjects, after excluding those with treated hypertension, treated dyslipidaemia, known diabetes, cardiovascular or renal disease, still had a high prevalence of unknown hypertension, dyslipidaemia and impaired glucose metabolism.

Table 1 shows the basic characteristics and cardiovascular risk factors of the cohort.

**Table 1:** Basic characteristics and metabolic risk factors of 1,191 apparently healthy subjects.

Parameters	N	%
Male gender	998	83.8
Unknown hypertension	279	23.4
Unknown hypercholesterolemia	86	7.2
Unknown IGT/diabetes	150	13.3
Obesity (BMI>30 kg/m^2^)	160	13.5
Abdominal adiposity (%)	168	16.0
No physical activity	491	49.2
Current smoking	370	32.4
Normoalbuminuria	183	15.4
Microalbuminuria	50	4.2
Macroalbuminuria	5	0.4

Unknown impaired glucose tolerance (IGT)/diabetes: plasma glucose ≥5.6 mmol/L. Hypercholesterolemia: serum cholesterol >6.5 mmol/L. Hypertension: RR ≥140 and/or 90 mmHg.

**Figure 1:**
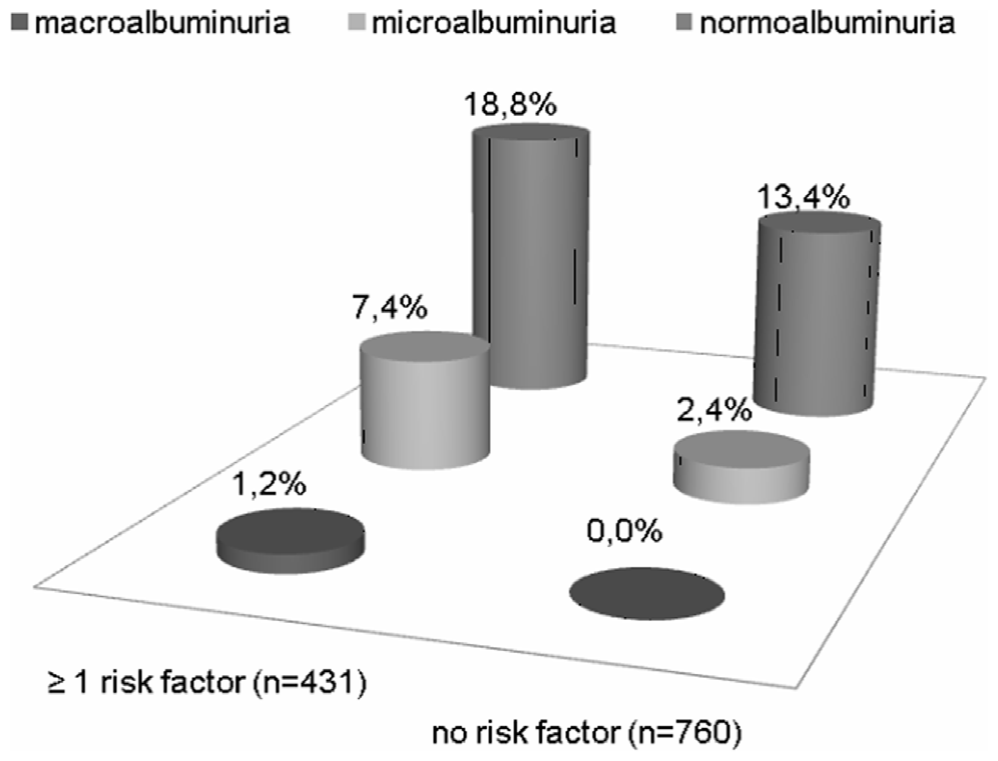
The prevalence of albuminuria in subjects with at least one vs. none risk factors.

Almost all subjects (98.9%) had an estimated GFR higher than 60 ml/min/1.73 m^2^ and none had an estimated GFR lower than 50 ml/min/1.73 m^2^. As expected, the cohort was rather young (age 38.3±9.7 years, range 17–64). Albumin was detected in the urine of one fifth. The large majority of albuminuric subjects had normoalbuminuria, fewer subjects had microalbuminuria and macroalbuminuria was only rarely observed (table 1).Table 2 shows the distribution of some measured clinical and biochemical parameters in subgroups according to different levels of albuminuria. In a randomly selected cohort (n = 599), a multivariate ordinal regression model selected resting heart rate, plasma glucose and hypertension as significant independent predictors for presence of albuminuria at any degree (table 3). The continuous variables were dichotomized (0 or 1) as follows: resting heart rate ≥85 bpm (cutoff level according to the 90^th^ percentile); plasma glucose: ≥5.6 mmol/L (impaired glucose tolerance). Consequently, the prevalence of normoalbuminuria, microalbuminuria and macroalbuminuria in this randomly selected cohort, was higher in subjects with at least one risk factor (n = 206) than in subjects with no risk factors (n = 393), respectively: 21.4 vs 14.1% (n = 44 vs 58); 8.7 vs 3.1% (n = 18 vs 12) and 1.5 vs 0% (n = 3 vs 0), p<0.001. Our risk assessment was validated in the other randomly selected cohort (n = 592). The prevalence of normoalbuminuria, microalbuminuria and macroalbuminuria in this validation cohort, was also higher in subjects with at least one risk factor (n = 225) than in subjects with no risk factors (n = 367), respectively: 16.4 vs 12% (n = 37 vs 44); 6.2 vs 1.6% (n = 14 vs 6) and 0.9 vs 0% (n = 2 vs 0), p = 0.001. Table 4 shows the test characteristics for subjects with at least one risk factor, to identify normoalbuminuria, microalbuminuria and macroalbuminuria in both randomly selected cohorts. Because our risk assessment fitted quite well in both cohorts, we applied it in the complete population. The prevalence of normoalbuminuria, microalbuminuria and macroalbuminuria in the complete population, was higher in subjects with at least one risk factor (n = 431) than in subjects with no risk factors (n = 760), respectively; 18.8 vs 13.4% (n = 81 vs 102); 7.4 vs 2.4% (n = 32 vs 18) and 1.2 vs 0% (n = 5 vs 0), p<0.001 (figure 1).

**Table 2:** Metabolic risk factors in subjects with different level of albuminuria.

Albuminuria	no	Normo-	Micro-	Macro-
n = 1,191 (%)	953	183 (15.4)	50 (4.2)	5 (0.4)
Age (years)	38.2±9.6	38.7±10.1	38.8±10.7	39.0±9.1
Men (%)	799 (83.8)	147 (80.3)	47 (94)	5 (100)
Systolic blood pressure (mmHg)	126.9±13.8	129.8±14.1	132.6±18.3*	142.6±11.2
Diastolic blood pressure (mmHg)	77.4±9.9	79.9±10.6*	80.8±13.2	90±8.9*
Unknown hypertension	199 (20.9)	55(30.1)*	20 (40)**	5(100)**
Resting heart rate	69.5±10.3	72.6±11.3**	75.9±16.1**	78.8±3.7
Total cholesterol (mmol/L)	5.0±1.0	5.1±1.0	4.9±1.1	7.0±1.0**
Plasma glucose (mmol/L)	4.8±0.7	5.0±1.2*	5.0±0.9	5.2±1.1
Unknown IGT/diabetes (%)	103 (11.5)	34(19.3)*	12(24)*	1(20)
Body mass index (kg/m^2^)	25.9±3.9	25.5±4.3	25.3±5.3	27.5±2.7
Abdominal adiposity (%)	135(16.1)	21 (12.7)	11(25.6)	1 (25)
Serum uric acid (µmol/L)	315±71	315±77	339±77	482±119**
White blood cell count (10^9^/L)	7.0±1.9	7.2±1.9	7.9±2.3**	8.0±1.8
C-reactive protein (mg/L)	2.2±4.8	2.5±4.2	4.1±1.0	3.4±3.6
Current smoking (%)	283(31)	64 (35.6)	20 (40)	3 (60)

Unknown impaired glucose tolerance (IGT)/diabetes: plasma glucose ≥5.6 mmol/L.

Hypertension: RR ≥140 and/or 90 mmHg. *p<0.05, **p<0.01 versus no albuminuria.

**Table 3:** Multivariate regression analyses to predict different categories of albuminuria.

Random sample n = 599, R^2^ = 0.07	E	St error	P
Resting heart rate (beats per min)	0.026	0.009	0.006
Plasma glucose (mmol/L)	0.341	0.141	0.015
Unknown Hypertension	0.487	0.237	0.040
White blood cell count	0.079	0.053	0.132
Serum uric acid (µmol/L)	0.001	0.001	0.464
Total serum cholesterol (mmol/L)	−0.106	0.104	0.310

**Table 4:** Test characteristics to identify normo-, micro- and macroalbuminuria with at least one risk factor in randomly selected populations and in the complete “healthy” population.

Population	albuminuria	sensitivity	specificity	PPV	NPV	LR+	LR−
random cohort n = 599	Normo-	43	70	24	85	1.4	0.8
	Micro-	60	70	11	96	2.0	0.6
	Macro-	100	70	2	100	3.3	≈0
validation cohort n = 592	Normo-	46	65	18	88	1.3	0.8
	Micro-	70	65	8	98	2.0	0.5
	Macro-	100	65	1	100	2.9	≈0
complete population n = 1191	Normo-	44	67	21	86	1.3	0.8
	Micro-	64	67	9	97	1.9	0.6
	Macro-	100	67	1	100	3.0	≈0

PPV: positive predictive value; NPV: negative predictive value; LR+: positive likelihood and LR−: negative likelihood ratio.

We evaluated two strategies for screening albuminuria in our population, one strategy where a set of additional risk factors were screened as first line, with later screening for albuminuria only in subjects with at least one of those additional risk factors and an alternative strategy where albuminuria was screened as first line, and additional risk factors were measured only in those with albuminuria.

A strategy where only subjects with at least one modifiable risk factor (n = 431) were screened for albuminuria, would identify all subjects with macroalbuminuria (5/5), 64% of subjects with microalbuminuria (32/50), but less than half of those with normoalbuminuria (81/183), table 4 shows the corresponding likelihoods and predictive values.

An alternative strategy whereby subjects were first screened for presence of albuminuria, and additional cardiovascular risk factors were only measured in subjects positive for albuminuria (n = 238), would identify only 27% (118/431) of the subjects with additional and potentially modifiable cardiovascular risk factors. On the other hand, half of the subjects in this study with albuminuria (120/238, of which 102 had normal range albuminuria), had no additional cardiovascular risk factor at all.

Table 5 shows that subjects with no modifiable risk factor had also lower levels of risk factors which were not included in our screening model.

**Table 5:** Cardiometabolic profile in subjects with none versus one or more risk factors.

Risk factors	None	≥1	p
N	760	431	
Age	37.0±9.4	40.6±9.8	<0.001
Male gender (%)	604 (79.5)	394 (91.48)	<0.001
Resting heart rate (bpm)	67.7±8.3	74.9±13.1	<0.001
Systolic blood pressure (mmHg)	121.9±10.4	137.9±14.1	<0.001
Diastolic blood pressure (mmHg)	74.4±8.3	84.3±10.2	<0.001
Plasma glucose (mmol/L)	4.6±0.5	5.3±1.0	<0.001
White blood cell count (10*10^9^/L)	6.9±1.9	7.3±1.9	<0.001
Body mass index (kg^2^/m)	25.2±3.6	26.9±4.4	<0.001
Abdominal obesity (%)	72 (10.8)	96 (24.8)	<0.001
Obesity >30 kg/m^2^ (%)	71 (9.4)	89 (20.6)	<0.001
Total cholesterol (mmol/L)	4.9±0.9	5.3±1.0	<0.001
Serum uric acid (µmol/L)	305±70	336±77	<0.001
C-reactive protein (mg/L)	2.0±3.4	3.0±7.0	<0.001
Current smoking	225 (30.7)	145 (34.9)	0.130
No physical activity	282 (44.3)	209 (57.9)	<0.001

## Discussion

Our data indicate that albuminuria, unknown hypertension and impaired glucose metabolism are quite prevalent findings in an apparently healthy population. When present, albuminuria was mostly measured in the normal range, and was frequently found in subjects without other modifiable risk factors, making its relevance as a predictor of outcome questionable. A screening strategy for albuminuria starting from assessment of simple and easy to obtain risk factors, such as resting heart rate, blood pressure and plasma glucose level identified subjects at risk for micro- and macroalbuminuria in a more effective way than a strategy of screening a healthy population for albuminuria alone.

There is little debate that screening for albuminuria should be performed in patients with diabetes and/or hypertension, where early intervention can slow down deterioration of renal function. Whether it should also be performed in the general population remains ambigiuous [12], [18], [19]. There is heated debate whether screening the healthy population for presence of albuminuria is fulfilling all conditions requested to define a successful screening program [13].

A first request is that the screened factor either relates to an important health risk, or is prevalent in the population. In our healthy population, the prevalence of albuminuria was 20%. However, many cases had normoalbuminuria without additional risk factors. Those in favor of screening the general population argue that an urinary albumin excretion >5 µg/min is related with increased risk of cardiovascular morbidity and mortality [20], [21], [22]. In some of these studies, an adjustment for presence of hypertension and diabetes was performed, indicating that albuminuria is an independent risk factor on top of diabetes and hypertension, but it is not clear whether the increased risk was also present in those with normal blood pressure and glucose levels [21]. In the PREVEND cohort, hypertension and diabetes were not actually assessed objectively, but were based on “self declared” status [23]. In our study, the prevalence of unknown hypertension and impaired glucose levels was high, even after excluding subjects with known risk factors, so most likely also in the PREVEND cohort a substantial part of “false negatives” for hypertension and impaired glucose tolerance are present. Accordingly, the definition of “healthy population” in the PREVEND cohort is probably not correct, making also the recommendation to screen the healthy population incorrect, as most likely a substantial number of subjects in this “healthy cohort” would have hypertension or elevated glucose levels if these would have been measured. In other studies restricted to non-diabetic and non-hypertensive subjects, although the increased risk for renal disease in those with microalbuminuria seems dramatic, the absolute risk remains low, with less than 0.1% of persons with microalbuminuria ending up on renal replacement therapy, or 0.6% developing cardiovascular disease over an 8 year period [9], [24]. In the PREVEND study, the relation between albuminuria and decline of renal function in subjects without known risk factors, was only observed in those individuals with macroalbuminuria [9]. As mentioned, in our study, all subjects with macroalbuminuria would be detected if only subjects with more than one risk factor were screened, as none of the subjects without risk factors (the truly healthy population) had macroalbuminuria. In our cohort, the prevalence of microalbuminuria, in subjects without risk factors, was 2.4%. A similar figure was found in a New Zealand study (2.0%) in subjects without diabetes, impaired glucose tolerance, hypertension or dyslipidaemia [25]. In the Copenhagen City Heart Study, the prevalence of microalbuminuria was also 2.0% in subjects without any feature of the metabolic syndrome, and these subjects had no increased risk for cardiovascular disease and death, suggesting that microalbuminuria by itself might not be an independent determinant of outcome without presence of associated risk factors [26]. It can be that this microalbuminuria is the equivalent of “exertional”[27] or “orthostatic” albuminuria.

Another request for a screening program to be meaningful and effective, is that risk factors should be modifiable. As a prospective trial to test the hypothesis that medical management of microalbuminuria affects patient-centered events independent of blood pressure reduction is still lacking [28], screening for microalbuminuria in subjects without measured additional risk factors, appears not to be justified from a general health care perspective.

In our cohort of apparently healthy subjects, the likelihood of having albuminuria was related to the well established and potentially modifiable risk factors blood pressure and plasma glucose level, but also to resting heart rate. Two thirds of our cohort had none of these risk factors. Table 5 shows that those subjects had also much lower levels of other cardiovascular risk factors not included in our risk score. Nevertheless, 13.4% of these subjects had normoalbuminuria, but none had macroalbuminuria. Most likely, these subjects have thus a very low absolute risk for cardiovascular or renal disease, and the potential benefit of a treatment should be considered very low. Consequently, a strategy of screening only in those with at least one risk factor would miss only few potentially relevant cases, at the same time avoiding many “false positives”, who compose nearly 50% of albuminuric subjects when unrestricted screening for albuminuria is performed first. However, testing for albuminuria in subjects with additional cardiovascular risk factors is warranted, as a more aggressive treatment can be defended in subjects with additional risk factors in presence of albuminuria compared to those with risk factors but without albuminuria.

There is substantial evidence that reduction of blood pressure, decreases the progression of renal disease and reduces cardiovascular events [29], [30], [31]. Disturbed glucose metabolism, as indicated by increased plasma glucose levels, is a condition with increased risk for the development of overt diabetes. Life style modification, and the use of drugs such as metformin and acarbose can slow down progression to overt diabetes and/or cardiovascular disease in these subjects [32]. The association of resting heart rate with albuminuria was previously mentioned [33], [34]. An explanation could be that a high resting heart rate may cause mechanical stress that might contribute to renal endothelial dysfunction leading to albuminuria [35]. Previous reports mentioned that tachycardia as a sign of sympathetic overactivity, is an independent risk factor for chronic kidney disease, cardiovascular and noncardiovascular mortality, even in an apparently healthy population [34], [36], [37], [38]. Reduction of sympathetic overactivity by regular physical activity and smoking cessation appear to be beneficial in this patient group [39]. Carvedilol appears to reduce proteinuria to a higher degree than expected by the blood pressure lowering effect alone in patients with hypertension, but it is unclear whether this effect is due to the reduction in heart rate modification or to genuine metabolic effects [40].

A strength of this study is the nearly 100% participation rate of a relatively young and apparently healthy occupational population. A limitation is that we only measured urinary albumin to creatinine ratio at one occasion, guidelines recommend to have at least two positive albumin to creatinine ratio's in three consecutive first morning urine samples before labeling a person with microalbuminuria[41]. Furthermore, the prevalence of microalbuminuria was underestimated if albumin to creatinine ratio was measured in morning urine samples [42], while an overestimation was observed if random samples were obtained [43].Thus, the association between subjects with albuminuria and additional risk factors could be confounded by measuring the urinary albumin to creatinine ratio at only one random occasion.

In conclusion, our data provide evidence to support the concept that screening for albuminuria should only be performed in subjects with additional and potentially modifiable risk factors, and that this strategy is more beneficial than screening the general population. We identified 3 parameters that are easy and cheap to obtain: blood pressure, plasma glucose and resting heart rate to identify subjects in whom further assessment of presence of albuminuria might be relevant.
